# Supplementary material for: The genome sequence of the chocolate mining bee, Andrena scotica Perkins, 1916 (Hymenoptera: Andrenidae)
Source: Wellcome Open Res. 2026 Mar 18;11:170. [Version 1] doi: 10.12688/wellcomeopenres.26130.1 (PMC13058575; doi:10.12688/wellcomeopenres.26130.1)
Supplement: Peer review history

# Reviewer report: Reviewer response for version 1

**DOI:** 10.21956/wellcomeopenres.28777.r151128

**Published:** 2026-04-07

**Authors:** Rodolpho S. T. Menezes

**Affiliations:** 1 Universidade Estadual de Santa Cruz (Ringgold ID: 74361), Ilhéus, State of Bahia, Brazil

The data note by Crowley et al. presents a genome assembly for the bee *Andrena scotica* Perkins, 1916 (Hymenoptera: Andrenidae). The assembled genome has a total length of 446.96 megabases. This genomic resource represents a valuable contribution to future comparative genomics studies.

 Overall, the methodology is robust and clearly described, the figures are well constructed, and the manuscript is well written.

 However, the background section would benefit from further development. In particular, the authors should expand on the broader significance of this study and provide a more detailed comparison of their results with those from other bee genomes, including species within the genus *Andrena*.

Are sufficient details of methods and materials provided to allow replication by others?

Yes

Is the rationale for creating the dataset(s) clearly described?

Yes

Are the datasets clearly presented in a useable and accessible format?

Yes

Are the protocols appropriate and is the work technically sound?

Yes

Reviewer Expertise:

Phylogenomics, Cytogenetics, and phylogeography.

I confirm that I have read this submission and believe that I have an appropriate level of expertise to confirm that it is of an acceptable scientific standard.

# Reviewer report: Reviewer response for version 1

**DOI:** 10.21956/wellcomeopenres.28777.r151125

**Published:** 2026-04-01

**Authors:** Eduardo Luís Menezes de Almeida

**Affiliations:** 1 Universidade Federal de Viçosa, Minas Gerais, Brazil

General comments:

 The data comprises a high-quality chromosome level assembly of *Andrena scotica* as part of the Darwin Tree of Life project. The methods and assembly data is well presented and consistent. Few points remain as described below.

 Specific comments:

 Background:

 The authors provide a good background for *Andrena* bees. I suggest also adding a brief paragraph regarding other *Andrena *genome sequences (if available) and common genetic characteristics (if available) like chr number, ploidy, genome size…, which were might also have been expected along the sequencing effort.

 Genome sequence report:

 Figure 2: I could not recover the repeat content in this figure. Could the authors elaborate on that?

 Assembly statistics: What could be responsible for the difference in size between the assembled genome and the prediction by GenomeScope2?

Are sufficient details of methods and materials provided to allow replication by others?

Yes

Is the rationale for creating the dataset(s) clearly described?

Yes

Are the datasets clearly presented in a useable and accessible format?

Yes

Are the protocols appropriate and is the work technically sound?

Yes

Reviewer Expertise:

I work with bioinformatics and computational biology, including genomics, transcriptomics, and metabolic modeling of prokaryotes and eukaryotes.

I confirm that I have read this submission and believe that I have an appropriate level of expertise to confirm that it is of an acceptable scientific standard.
